# A *Ganoderma-*Derived Compound Exerts Inhibitory Effect Through Formyl Peptide Receptor 2

**DOI:** 10.3389/fphar.2020.00337

**Published:** 2020-03-24

**Authors:** Huirong Wang, Xingrong Peng, Yunjun Ge, Shuo Zhang, Zhenyi Wang, Yu Fan, Wei Huang, Minghua Qiu, Richard D. Ye

**Affiliations:** ^1^Institute of Chinese Medical Sciences, State Key Laboratory of Quality Research in Chinese Medicine, University of Macau, Macau, Macau; ^2^Department of Biology, Southern University of Science and Technology, Shenzhen, China; ^3^Kunming Institute of Botany, Chinese Academy of Science, Kunming, China; ^4^School of Pharmacy, Shanghai Jiao Tong University, Shanghai, China; ^5^Hefei National Laboratory for Physical Sciences at the Microscale and School of Life Sciences, University of Science and Technology of China, Key Laboratory of Structural Biology, Chinese Academy of Sciences, Hefei, China; ^6^Kobilka Institute of Innovative Drug Discovery, School of Life and Health Sciences, The Chinese University of Hong Kong, Shenzhen, China

**Keywords:** formyl peptide receptors, *Ganoderma*, anti-inflammatory, chemotaxis, superoxide, fluorescence resonance energy transfer

## Abstract

Formyl peptide receptors (FPRs) are G protein-coupled receptors (GPCRs) widely expressed in neutrophils and other phagocytes. FPRs play important roles in host defense, inflammation, and the pathogenesis of infectious and inflammatory diseases. Because of these functions, FPRs are potential targets for anti-inflammatory therapies. In order to search for potentially novel anti-inflammatory agents, we examined *Ganoderma* (Lingzhi), a Chinese medicinal herbs known for its anti-inflammatory effects, and found that compound 18 (C18) derived from *Ganoderma cochlear* could limit the inflammatory response through FPR-related signaling pathways. Further studies showed that C18 could bind to FPR2 and induce conformation change of the receptor that differed from the conformational change induced by the pan-agonist, WKYMVm. C18 inhibited at the receptor level and blocked WKYMVm signaling through FPR2, resulting in reduced superoxide production and compromised cell chemotaxis. These results identified for the first time that a *Ganoderma*-derived component with inhibitory effects that acts through a G protein-coupled receptor FPR2. Considering its less than optimal IC_50_ value, further optimization of C18 would be necessary for future applications.

## Introduction

Formyl peptide receptors (FPRs) are cell surface pattern recognition receptors (PRRs) that belong to the evolutionarily conserved family of G protein-coupled receptors (GPCRs). They are widely expressed in circulating blood granulocytes (de Paulis et al., 2004; Migeotte et al., 2006; Svensson et al., 2007), especially neutrophils, the most abundant type of circulating leukocytes (Dorward et al., 2015). Although three subtypes of FPRs have been identified in human (FPR1, FPR2, and FPR3), only FPR1 and FPR2 are expressed on neutrophils and play a vital role in innate immunity (Murphy et al., 1992; Ye et al., 2009). The primary role of the FPRs is to recognize N-formylated peptides of protein fragments from bacteria and mitochondria, and induce pro-inflammatory responses such as chemotaxis, superoxide generation, and degranulation. These bactericidal functions contribute to the clearance of invading microbes and removal of tissue debris (Ye et al., 2009). In addition, FPRs also play important roles in various inflammatory diseases (Weiss and Kretschmer, 2018). For example, inhibition of mouse FPRs attenuates obesity-linked inflammation and leads to increased glucose tolerance and insulin levels in obese mice (Claria et al., 2012; Wollam et al., 2019). Activation of mouse FPR2 alleviates scleroderma-associated fibrosis, suppresses inflammation, and attenuates joint injury in rheumatoid arthritis mouse model (Kao et al., 2014; Odobasic et al., 2018; Park et al., 2018; Park et al., 2019). Moreover, FPRs also have been implicated in various cancers. FPR1was found in colorectal, gastric, and breast cancers (Cheng et al., 2014; Prevete et al., 2015; Baracco et al., 2016; Li et al., 2017; Prevete et al., 2017). FPR2 expression was associated with poor prognosis by mediating chemotherapeutic drug resistance (Xiang et al., 2016; Xie et al., 2017; Su et al., 2018). Besides, FPR2 is also involved in attenuating HIV-1 infection by desensitizing other chemokine receptors (CCR5 and CXCR4) and suppressing IL-12 production in human monocytes (Braun et al., 2001; Li et al., 2001). Based on these important roles in inflammatory diseases, FPRs could be potential targets for antagonizing inflammatory responses in certain diseases including cancer.

The complex role in various diseases indicates FPRs are potential targets for therapeutic intervention. Therefore, searching for novel agonists and antagnosits of FPRs has drawn significant attention. The FPR subfamily has a variety of structurally diverse ligands, including natural peptides and synthetic non-peptide compounds. Compared with natural peptides, small molecule comounds are more stable to serve as potentially therapeutic agents (He and Ye, 2017). Although numerous small molecule compounds with inhibitory effects have been found through screening of combinatorial compound libraries, very few were fully characterized with high potency (*K*i < 10 µM) in the literatures (Schepetkin et al., 2014; He and Ye, 2017). Traditional Chinese medicine (TCM) has been explored in search for novel FPR antagonists, based on its long-term medical practice (Yuan et al., 2016). In this study, *Ganoderma* (Lingzhi) was chosen because it has been used extensively in Asian countries for more than 2,000 years, due to its various pharmacological effects, including immunomodulation, antibacterial, anticancer, antioxidant, and antiviral activities (Gao et al., 2003; Sliva, 2004; Yuen and Gohel, 2005; Joo et al., 2008; Sanodiya et al., 2009; Ma et al., 2011; Xu et al., 2011). *Ganoderma* is rich in active compounds, including triterpenoids, fatty acids, polysaccharides, peptides, and other chemicals (Sanodiya et al., 2009; Peng and Qiu, 2018), and that has led to the possibility of identifying FPR agonists and antagnosits.

**Figure 1:**
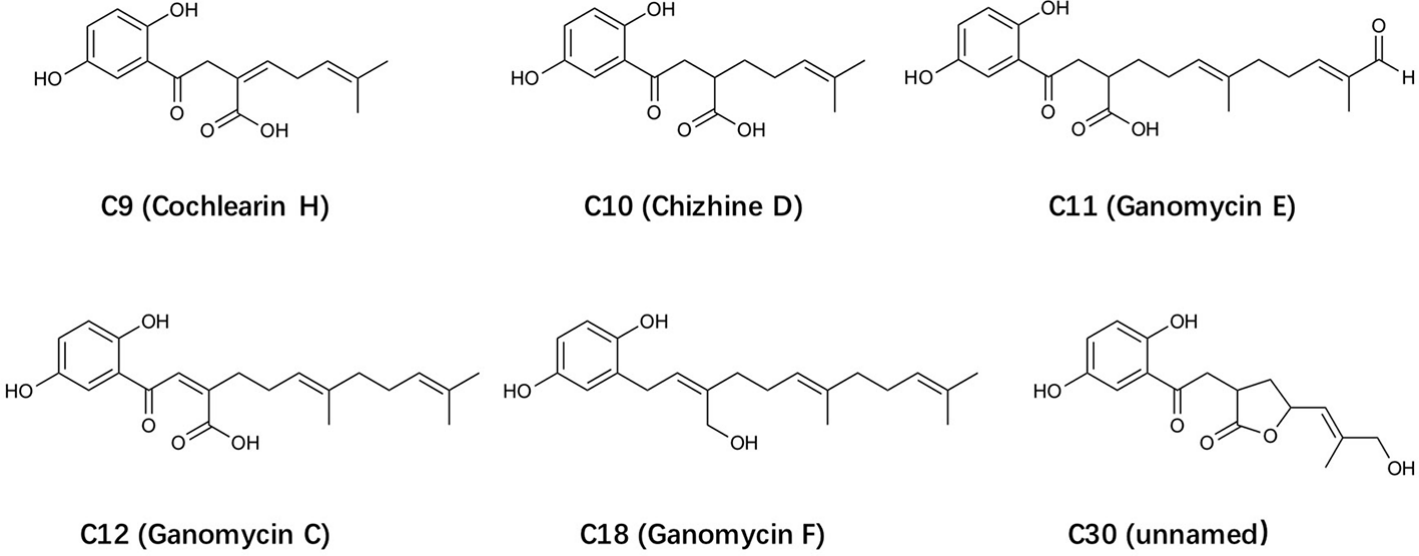
The structure of selected *Ganoderma-*derived compounds.

In this study, 34 *Ganoderma*-derived compounds that were available in our collection were subjected to initial screening using FPR2-dependent superoxide generation assay and degranulation assay. Among these triterpenoids and meroterpenoids, C18 was identified to have strong inhibitory activities. C18 and 5 other structurally similar compounds (**Figure 1**), all *Ganoderma* meroterpenoids (GMs) (Peng and Qiu, 2018), were selected for further studies. (**Figure 1**). C18, was found to display significant inhibition in several FPR-mediated functional assays, but had no effect on C5a receptor and PKC-mediated signaling pathways. To assess the structure-activity relationship, FLAsH-based fluorescence resonance energy transfer (FRET) detection and molecular docking analysis were performed. The results demonstrated that C18 could inhibit FPR-mediated pro-inflammatory response by targeting FPR2. In short, our work demonstrated the *in vitro* inhibitory effects of a novel *Ganoderma*-derived compound through FPR2, further revealing its detailed mechanism with competitive binding assay and FRET detection assay, and finally show its interaction with FPR2 by molecular docking analysis. These results suggest that C18 may be a naturally active component and exert its inhibitory effects through FPR2.

## Materials and Methods

### Materials

N-Formyl-Met-Leu-Phe (fMLF, ≥97% purity), Phorbol-12-myristate-13-acetate (PMA), Dimethyl sulfoxide (DMSO), isoluminol, Horseradish peroxidase (HRP), Albumin from bovine serum (BSA), P- Nitrophenyl-N-acetyl-β-D-glucosamide, and cytochalasin B were purchased from Sigma (St. Lous, USA). WKYMVm (m=D-Met; ≥95% purity) was synthesized by Syn High Quality Peptide (Shanghai, China). Fluorescent peptide, WK(FITC)YMVm (≥95% purity) was synthesized and conjugated by ChinaPeptides CO., Ltd (Shanghai, China). FLIPR Calcium five assay kit was purchased from Molecular Devices (Sunnyvale, United States). The RT-PCR kit was purchased from Takara Bio (Kusatsu, Japan). FBS, Hank's balanced salt solution (HBSS) with or without Ca^2+^ and Mg^2+^, Hoechst, other cell culture medium, and Alexa Fluor ^®^488 goat anti-rabbit IgG (H+L) were purchased from Thermo Fisher Scientific (Waltham, USA). Alexa Fluor ^®^647 mouse anti-human fMLP receptor, Clone 5F1(RUO) was purchased from BD Bioscience (New Jersey, USA), and FPR2 antibody was purchased from Novus Biologicals (Colorado, USA). The fruiting bodies of *Ganoderma cochlear* (Ganodermataceae) were purchased from the Traditional Chinese Medicine Market at Luosiwan International Trade City (Kunming, China). A specimen (No. 13071501) was deposited in the Herbarium of the Department of Taxonomy, Kunming Institute of Botany, Chinese Academy of Sciences.

### Compounds Preparation

As shown in **Supplementary Figure 1**. *G. cochlear* (68 kg) mushrooms were chipped and extracted with 95% ethanol (EtOH, 120 L) under reflux three times at 60°C, each for 3 h. The combined ethanol extracts were evaporated under reduced pressure. The residue was suspended in H_2_O (10 L) and extracted with ethyl acetate (EtOAc, 3 × 10 L) and *n*-Butanol (3 × 10 L), respectively. The volume of the combined EtOAc extracts was reduced to one-third under reduced pressure. The residue (11.5 kg) was fractionated by macroporous resin (D-101; MeOH/H_2_O, 50:50, 70:30, and 90:10, v/v): fractions I–III.

Fr. II-A (50 g) was taken from Fr. II (232 g) and was subjected to column chromatography (RP C-18, MeOH/H_2_O, 30:70→100:0, v/v) to get nine subfractions. Furthermore, Fr. II-A-2→Fr. II-A-9 were processed repeated using silica gel, Rp-C18, LH-20, and HPLC to yield 20 compounds including C9 and C10. Fr. II-B (50 g) was taken from Fr. II and was treated by column chromatography (CC silica gel, CHCl_3_/MeOH, 100:1, 80:1, 50:1, 20:1, and 5:1, v/v) to gain five subfractions (Fr. II-A-1 → Fr. II-A-5). By TLC analysis, Fr. II-A-3 and Fr. II-A-4 contain mostly triterpenoids and meroterpenoids. Thus, the above two subfractions were further separated using Rp-C18, Sephadex LH-20 (MeOH), preparative TLC (CHCl_3_–MeOH, v/v) and HPLC to obtain the additional compounds, including C11, C12, C18, and C30.

The molecular formula of testing active compound C18 and the negative control compound C12 were determined by HRESIMS and ^13^C-DEPT NMR at Kunming Institute of Botany, China, as previously described (Peng et al., 2016). These compounds were analysis by HPLC and determined to have a purity ≥ 95% (**Supplementary Figure 2**).

### Cell Cultures

Human promyeloblast leukemia HL-60 cells were cultured in RPMI 1640 medium supplemented with 10% heat-inactivated FBS, 100 Mg/ml streptomycin, 100 U/ml penicillin. For HL-60 differentiation (dHL-60), DMSO was added to a final concentration of 1.3% (v/v) and the cells were cultured for an additional 6 days. Rat basophils leukemia cells (RBL-2H3, ATCC^®^ CRL-2256™) were stably transfected with human FPR2 (RBL-FPR2 cells) and cultured in DMEM supplemented with 20% FBS, 100 Mg/ml streptomycin, 100 U/ml penicillin, and 250 Mg/ml G418 (He et al., 2000). COS^phox^ cells were cultured in DMEM supplemented with 10% FBS, 100 Mg/ml streptomycin, 100 U/ml penicillin, 0.5mg/ml G418, 0.8 mg/ml neomycin and 0.2 mg/ml hygromycin (Price et al., 2002).

### Calcium Mobilization Assay

RBL-FPR2 or RBL-FPR1 cells were cultured in black wall/clear bottom 96-well plate until the confluence reached about 90%. The cells were washed once with DMEM and incubated with FLIPR calcium-sensitive dye (Molecular Devices, Sunnyvale, CA) and different concentration of compounds to be tested or vehicle (0.1% DMSO) in HBSS/BSA for 60 min at 37°C with 5% CO_2_. The agonist (WKYMVm, 10 nM for screening and 10^-12^-10^-7^ M for dose-response curve; fMLF, 10^-9^-10^-6^ M for dose-response curve) was added and samples were read in a FlexStation III Multi-Mode Microplate Reader (Molecular Devices) with excitation wavelength at 488 nm and emission wavelength at 525 nm according to the manufacturer's protocol (He et al., 2013).

### Superoxide Generation Assay

Superoxide production of differentiated HL-60 cells (dHL60-6d, 1×10^5^ cells per well) was determined by isoluminol-ECL assay (Dahlgren and Karlsson, 1999), using 96-well, flat-bottom, white tissue culture plates (PerkinElmer Life Sciences, Boston, MA). dHL60 cells were harvested and washed once with 0.5% BSA/HBSS. Cells were then re-suspended with 0.5% BSA/HBSS buffer and incubated with or without compounds for 30 min, then added with 100 μM isoluminol and 40 U/ml HRP at 37°C for 5 min in the dark. Aliquots (200 Ml) of the cells were added into the 96-well plate, and chemiluminescence (CL) was eventually detected at 37°C with an EnVision Multilabel Plate Reader (PerkinElmer Life Sciences, Boston, MA). The CL counts per second (CPS) was continually recorded, at 16 s intervals, for 20 points before and 200 points after stimulation with 100 ng/ml PMA or 1 μM fMLF or WKYMVm. The relative level of superoxide anion produced was calculated based on the integrated CL during the first 15 min after agonist stimulation.

### Cell Degranulation Assay

For release of β-hexosaminidase, RBL-FPR2 cells were cultured in a 24-well plate for 24 h, or differentiated HL-60 cells for 6 days (dHL60-6d, 1×10^5^ cells per well), then treated with or without compounds for 1 h. Subsequently, cells were washed briefly and pre-incubated with 10 μM cytochalasin B in HBSS included 20 mM HEPES, pH 7.4, and 0.5% BSA (HBSS-HB) for 15 min on ice followed by 15 min at 37°C, as described in a previous publication (Nanamori et al., 2004). Then, cells were stimulated for 15 min with 1 μM WKYMVm and vehicle at 37°C before chilling on ice to terminate the degranulation reaction. The amount of secreted β-hexosaminidase was quantified by incubating 20 μl of supernatant with 10 μl of 1 mM p-nitrophenyl-N-acetyl-β-D-glucosamide in 0.1 M sodium citrate buffer, pH 4.5, at 37°C for 1 h in a 96-well plate. Then reaction was terminated by adding 200 Ml of 0.1M Na_2_CO_3_ and 0.1M NaHCO_3_, pH 10, and absorbance was determined at 405 nm in a Flex Station 3 Multi-Mode Microplate Reader (Molecular Devices). Total cellular β-hexosaminidase was determined with cell lysate in 0.1% Triton X-100.

### Cell Chemotaxis Assay

Agonist-induced migration of cells was assessed in a 24-well transwell chamber (Corning costar, Kennebunk, USA), as reported previously (Nanamori et al., 2004). In brief, dHL-60 cells (2×10^5^ cells per well) were pre-incubated with different concentrations of the compounds (10 nM–20 μM) for 30 min and then seeded the cells in the upper chamber (100 Ml), WKYMVm (1nM) was placed in the bottom well (600 Ml), which was separated from the lower compartment by a polycarbonate membrane filter with pore size of 5 μm. After incubation at 37°C for 2 h, the upper chamber was removed and the total number of cells in the bottom wells were counted by flow cytometry. Data were presented as chemotaxis index, which was the ratio of cells migrated toward agonists over the cells migrated toward medium. Checkerboard analysis was performed by adding 2×10^5^ dHL60 cells/well to the upper chamber, and serial dilutions of C18 were added to the upper chamber as well as the lower chamber. After 2 h, cells that migrated through the polycarbonate membrane were counted in the lower chamber.

### FLAsH-Based Fluorescence Resonance Energy Transfer (FRET) Detection

Using the methods described previously (Gaietta et al., 2002; Hoffmann et al., 2005), HEK-293 cells (ATCC^®^ CRL-1573) were cultured in 24-well plate on coverslips with poly-D-lysine treatment. Plasmid coding for FPR2-ICL3-ECFP (FPR2 protein with ECFP at the C-terminal and FLAsH-binding sequence in the third intracellular loop) was transiently transfected into the cells. Twenty-four hours after transfection, TC-FlAsH™ II In-Cell Tetracysteine Tag Detection Kit (Thermo Fisher Scientific, Waltham, MA USA) was used to label the modified FPR2 proteins according to the manufacturer's instructions. In brief, the cells were washed with HBSS buffer and incubated with FlAsH-EDT2 labeling reagent (500 nM FLAsH/EDT, 12.5 μM EDT, and 5.6 mM glucose in HBSS) for 1 h. The excess and nonspecifically bound FLAsH was removed by incubating the cells with BAL wash buffer (250 μM BAL in HBSS) for 10 min twice followed by another wash with HBSS buffer. The cells were then treated with or without the indicated compounds for 10 min.

The coverslips with the cells were mounted on glass slides and FRET signals were analyzed with a Leica TCS SP8 confocal microscope immediately. The fluorescent FPR2 proteins were excited by a 448-nm laser and two images representing FLAsH emission and ECFP emission, respectively, were taken simultaneously with a dichroic beam splitter D448/D514 under a 40× oil objective. The emission paths are 535 ± 15 nm (FLAsH) and 480 ± 20 nm (ECFP). FRET signals were calculated as ratios of FLAsH intensities to ECFP intensities from the correspondent two images. Five dots with 5 × 5 pixels each at the cell membranes were chosen to obtain the fluorescence intensities for each cell sample.

### Cell Morphology Observation

Stably transfected FPR2-RBL cells were seeded in 12-well plate with round coverslips for 24 h. Then the cells were treated with or without C18 (10 μM) for 30 min, and stimulated with WKYMVm (final concentration of 1 μM) for another 15 min at 37°C. Subsequently, remove cell medium and wash the cells once with PBS, then fixed cells with 4% Paraformaldehyde for 15 min at room temperature, remove fixative solution and washed twice with PBS, blocked with 5% BSA+PBS for 1 h, and then stained with rhodamine phalloidin (Cytoskeleton, USA) and Hoechst according to the manufacture's protocol. In brief, stain the cells with 100 nM rhodamine phalloidin, incubate at room temperature in the dark for 30 min wash the cell coverslips three times in PBS, and stain DNA for 5 min with of 2 Mg/ml Hoechst. Rinse the coverslips and invert on a drop of anti-fade mounting media on a glass slide and seal each side with nail polish. Coverslips with the cells were mounted on glass slides and cell images were taken immediately with a 40× oil objective. The images were analyzed by ImageJ 1.49U (Rasband, W.S., ImageJ, U. S. National Institutes of Health, Bethesda, Maryland, USA, https://imagej.nih.gov/ij/, 1997-2018).

### Molecular Docking Analysis of the Binding Interaction of C18 With FPR2

Because the crystal structures of FPRs are not currently available, the structure models of FPR2 was obtained from Swiss-model server which was based on C5a receptor (PDB code: 5o9hA) because of its higher similarity (34.5%) and resolution (2.7Å) (Robertson et al., 2018; Waterhouse et al., 2018). The initial conformations of ligands were generated by ChemBio 3D (PerkinElmer). Hydrogens were added by Autodock Tools and molecular docking was performed by Autodock Vina (Trott and Olson, 2010). The search box was set as 46 Å × 34 Å × 60 Å for FPR2. The best conformation was refined with energy minimization and analyzed with PyMOL Molecular Graphics System (Version 2.0 Schrödinger, LLC).

### Competitive Binding Assays

RBL-FPR2 or RBL-FPR1 cells (4×10^4^ cells per well) were harvest and washed twice with buffer (HBSS supplemented with 20 mM HEPES, pH 7.5, and 0.1% BSA). These methods prepared as described (He et al., 2013). The competitive binding assays is used to measure relative affinity of WKYMVm and compound C18, in which a fixed concentration of WK (FITC) YMVm (50 nM) or fMLFIIK-FITC (100 nM) was added, and then added increasing concentrations of competitors and incubated for 1 h on ice. The mean fluorescence intensity (MFI) values were measured with flow cytometry.

### Cell Viability Analysis

HEK-293 cells were seeded in 96-well plate for 24 h and then treated with different concentrations of compounds (1 μM–50 μM) for another 24 h. The viability of cells was then measured with cell counting kit-8 (CCK8 kit, Dojingdo, Japan) according the manufacturer's procedures.

### Statistical Analysis

Data were shown as mean ± standard deviation (SD) from at least 3 independent experiments. Statistical analyses were performed using GraphPad Prism (Version 6.0, La Jolla, CA), IC_50_ values from each assay were calculated from dose response curve that were fitted by non-linear regression analysis. The differences of screening results were analyzed *via* one-way ANOVA with Dunnett's multiple comparison test. Other samples were analyzed with Student's *t*-test, and probability values of 0.05 or less were considered statistically significant.

## Results

### Screening the Active Components of *Ganoderma* for Inhibitory Properties

To identify components that regulate inflammation, FPR ligand-induced superoxide generation and cell degranulation assays were used for initial screening (**Supplementary Figure 3**). After exclusion of the cytotoxicity (data not shown), six compounds with similar structure (**Figure 1**) were selected from a pool of 34 *Ganoderma*-derived compounds, by using differential HL-60 (dHL60) cells. Since neutrophils have to be freshly isolated from human subjects and their lifespan is short, dHL60 cells were used as a substitution model for neutrophils in this study. HL-60 is a human promyelocytic leukemia cell line that acquires neutrophil-like properties when differentiated with 1.3% DMSO (Hauert et al., 2002). The preliminary data confirmed that both FPR1 and FPR2 were highly expressed in dHL60-6d cells, and the cells could efficiently generate superoxide upon stimulation with fMLF or WKYMVm (**Supplementary Figure 4**).

**Figure 2:**
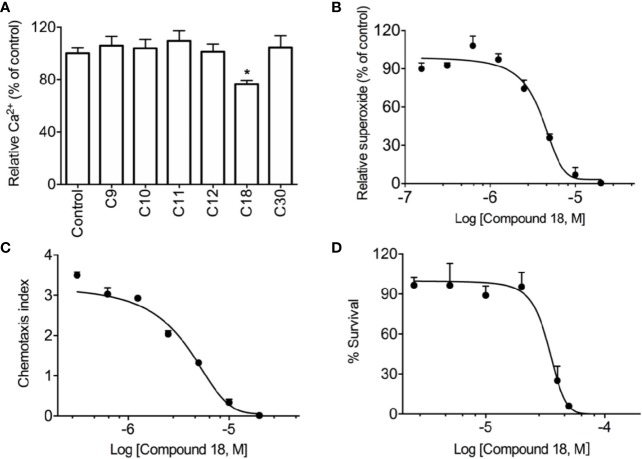
Screening results of *Ganoderma*-derived compounds. RBL-FPR2 cells were seeded until the confluence reached nearly 90%, and then incubated with FLIPR calcium-sensitive dye and 10 μM of *Ganoderma-*derived compounds for 60 min at 37°C. After that, the cells were simulated with 10 nM WKYMVm and relative fluorescence unit (RFU) was recorded. The results show that only C18 could inhibit calcium mobilization in stably transfected RBL-FPR2 cells **(A)**. Differentiated HL-60 cells (dHL60-6d, 1×10^5^ cells per well) were pre-incubated with C18 (10 nM–20 μM) for 30 min, 37°C. Chemiluminescence count per second (CPS) was continually recorded after stimulation with 1 μM WKYMVm, as described in *Methods*. The results show that C18 displayed significant inhibition of superoxide generation, with an IC_50_ of 4.0 μM **(B)**. dHL-60 cells (2×10^5^ cells per well) were pre-incubated with different concentrations of C18 (10 nM–20 μM) for 30 min and then seeded to transwell plate. WKYMVm (1nM) was placed in the bottom well (600 μl), which was separated from the lower compartment by a polycarbonate membrane filter with pore size of 5 μm. After incubation at 37°C for 2 h, the total number of migrate cells were counted. Data were presented as chemotaxis index, which represents the ratio of cells migrated toward agonists over the cells migrated toward medium. The results show that C18 has an IC_50_ of 3.8 μM for cell chemotaxis **(C)**. HEK-293 cells were seeded in 96-well plate for 24 h and then treated with different concentrations of C18 (1 μM–50 μM) for another 24 h. The viability of cells was then measured with CCK8 kit (Dojingdo, Japan) according the manufacturer. The IC_50_ value (32.9 μM) for C18 was then calculated **(D)**. Data are shown as Mean ± SD of three independent experiments. ^*^P < 0.05 versus vehicle-treated cells (control group). RBL, Rat basophils leukemia cells; FPR2, formyl peptide receptor 2.

The initial screening revealed that among these 6 compounds, cochlearin H (C9) (Peng et al., 2018), chizhine D (C10) (Luo et al., 2015), C30 (unpublished), and especially ganomycin F (C18) (Peng et al., 2016) significantly inhibited WKYMVm-induced superoxide production in dHL60 cells (**Supplementary Figure 3A**). Meanwhile, C18 also has distinct inhibition on WKYMVm-induced cell degranulation in dHL60 cells (**Supplementary Figure 3B**). This compound was selected for further analysis because it was the only compound in the small group that also inhibited WKYMVm-induced Ca^2+^ mobilization (**Figure 2A**), although C18 alone could not induce Ca^2+^ mobilization even at micromolar concentrations (**Supplementary Figure 5**). Furthermore, C18 dose-dependently inhibited superoxide generation (**Figure 2B**) and cell migration (**Figure 2C**) while having low cell toxicity (**Figure 2D**). A checker-board analysis was performed and it was confirmed that the inhibition in chemotaxis induced by C18 was due to the concentration difference between the upper chamber and lower chamber (**Supplementary Table 1**).

### Identification of the Pharmacological Target of C18

**Figure 3:**
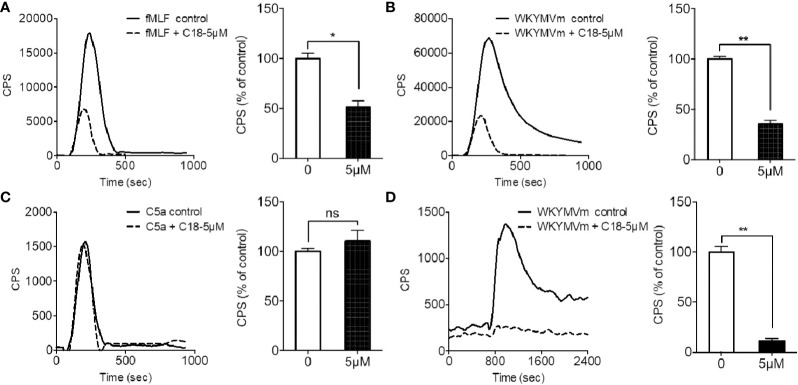
Effects of *Ganoderma*-derived C18 on different agonist-induced response. Differentiated HL-60 (dHL60-6d, 1×10^5^ cells per well) were pre-incubated with 5 μM compound C18 for 30 min, 37°C. Chemluminescence count per second (CPS) was continually recorded after stimulation with 1 μM fMLF **(A)**, 1 μM WKYMVm **(B)**, and 1 μM C5a **(C)**. The results show that fMLF and WKYMVm-induced superoxide generation were significantly inhibited by C18 at 5 μM, but C5a-induced superoxide generation was not inhibited by C18. To further confirm whether C18 targets on FPR2, co-transfected COS^phox^ cells with FPR2, this engineered cell model could generate superoxide only depend on FPR2. The cells were collected and incubated with or without 5 μM of C18 for 30 min before stimulating with 1 μM of WKYMVm. The results show that superoxide generation is nearly completely suppressed by 5 μM of C18 **(D)**. Data are shown as Mean ± SD of three independent experiments. ^*^P < 0.05; ^**^P < 0.01 versus control group cells (without C18). fMLF, N-Formyl-Met-Leu-Phe; FPR2, formyl peptide receptor 2; NS, No significance..

To further investigate the action mechanism of C18, the inhibitory effects on several GPCR-dependent pathways were compared, including FPR1, FPR2, and the complement component 5a (C5a) receptor. In addition, phorbol-12-myristate-13-acetate (PMA), an analogue of diacylglycerol (DAG), can directly stimulates protein kinase C (PKC) for PKC-dependent superoxide generation (Mellor and Parker, 1998) and was therefore included as a GPCR-independent agonist. As shown in **Figure 3**, C18 was selective for FPR-mediated superoxide generation induced by fMLF (**Figure 3A**) and WKYMVm (**Figure 3B**), as superoxide generation induced by C5a (**Figure 3C**) and PMA (**Supplementary Figure 6**) was not significantly inhibited by 5 µM C18. The results were further confirmed in genetically engineered COS^phox^ cells expressing FPR2 (Nie et al., 2010). Since COS^phox^ cells stably express gp91^phox^, p22^phox^, p67^phox^, and p47^phox^ and lack the hemopoietic specific proteins such as the FPRs, this cell model could be useful to generate high-level superoxide in a FPR2-dependent manner (Price et al., 2002). As shown in **Figure 3D**, C18 at 5 µM strongly inhibited WKYMVm-induced superoxide generation, similarly to the inhibitory effect seen in the dHL60 cells. Since the C5a receptor signaling mechanism is similar to that of the FPRs, these results indicate that C18 exerts its inhibitory effect at the receptor level of FPR.

### Investigation of the Effects of C18 on FPR-Mediated Cellular Functions

**Figure 4:**
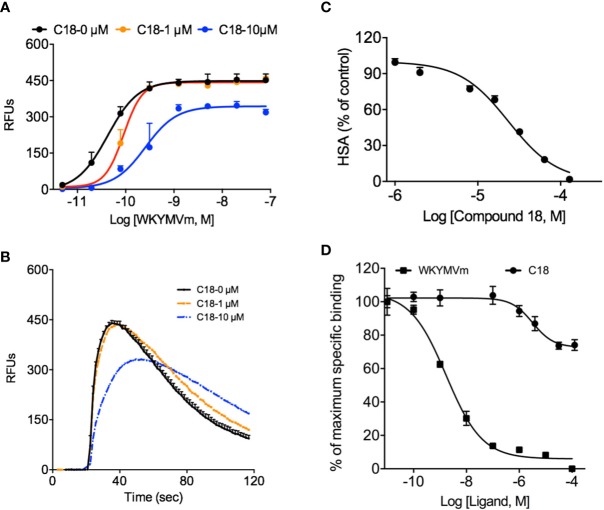
Effects of *Ganoderma*-derived C18 on other FPR-mediated cellular functions. RBL-FPR2 cells were seeded until confluence reached about 90%, and then washed once with DMEM and incubated for 60 min at 37°C with FLIPR calcium-sensitive dye and C18 at different concentrations (0, 1, 10 μM). The different concentration of agonist (WKYMVm, 10^-12^ - 10^-7^ M) was added robotically and relative fluorescence unit (RFU) was recorded. The results indicate that C18 increased EC_50_ of Ca^2+^ mobilization in stably transfected RBL-FPR2 cells **(A)** and the original Ca^2+^ response curve that stimulated with 10 nM WKYMVm of different concentration of C18 was compared in **(B)**. Pre-incubation RBL-FPR2 cells with or without 10 μM C18 for 60 min reduced 1 μM WKYMVm induced β-hexosaminidase (HSA) release, with an IC_50_ of 22.3 μM **(C)**. RBL-FPR2 cells (4×10^4^ cells per well) were harvest for competitive binding assay with a fixed concentration of WK(FITC)YMVm (50 nM) and increasing concentrations of competitors. After incubation for 1 h on ice, the mean fluorescence intensity (MFI) values were measured with flow cytometry. The binding curve indicates that higher concentration of C18 could partially compete with fluorescently labeled WKYMVm **(D)**. Data are shown as Mean ± SD from representative samples taken from three independent experiments, each with similar results. RBL, Rat basophils leukemia cells; FPR2, Formyl peptide receptors.

As shown in **Supplementary Figure 7**, besides superoxide generation and chemotaxis assays, formyl peptide receptors (FPRs) mediate other cellular responses such as Ca^2+^ mobilization, and degranulation during innate immune response. Based on the previous results that C18 exerts its inhibitory effects at receptor level, a series of functional assays were performed using RBL-FPR2 cells, because C18 only compete with ligand binding to FPR2 but not FPR1 (data not shown), as discussed below. As showed in **Figure 4**, pre-incubation with different concentrations of C18 caused a right-shift of the EC_50_ values in Ca^2+^ mobilization assays, increasing from 42.3 pM with 1 μM of C18 to 255.8 pM with 10 μM of C18 (**Figure 4A**), with concomitant reduction in the maximum response (**Figure 4B**). Meanwhile, C18 exhibited inhibitory effects in degranulation assays in a dose-dependent manner (**Figure 4C**). To investigate the molecular mechanism, competitive binding assays were performed to verify whether C18 could compete for active site of FPR2, and the results show that C18 could not compete effectively with WKYMVm-FITC, but could compete partially at higher concentrations (IC_50_: 3.2 μM). In comparison, WKYMVm could compete with its fluorescein labeled ligand with a higher affinity (IC_50_: 1.7 nM;) (**Figure 4D**). As will be discussed below, C18 may have an allosteric effect on FPR2 at higher concentrations.

### Observation of the Changes of C18 Targets on FPR-Mediated Cell Morphology

**Figure 5:**
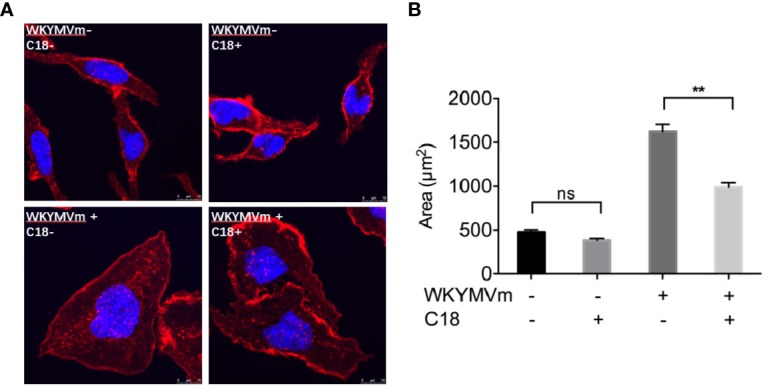
C18 inhibited WKYMVm-induced cell spreading. FPR2-RBL cells were seeded on round coverslips and placed in a 12-well plate for incubation for 24 h. The cells were then incubated with or without C18 (10 μM) for 30 min, followed by stimulation with WKYMVm (final concentration of 1 μM) for 15 min at 37°C. After fixing the cells with 4% paraformaldehyde, rhodamine phalloidin (100 nM) was added for staining of actin and the cells, then incubated the cells at room temperature in the dark for 30 min. The nuclei were stained for 5 min with 2 Mg/ml Hoechst. The mounted coverslips were viewed immediately with a 40× oil objective **(A)**. Area of each cells was measured by ImageJ **(B)**. Data are shown as Mean ± SD of five cells from representative samples taken from 3 independent experiments, each with similar results. ^**^P< 0.01 versus control group cells (without C18). FPR2, Formyl peptide receptors; RBL, Rat basophils leukemia cells; NS, No significance.

Due to its inhibitory effects on almost entirely FPR-mediated functional assays, cell morphological changes caused by C18 have attracted our attention. As shown in **Figure 5**, 10 μM; C18 was pre-incubated with RBL-FPR2 cells for 30 min, then stimulated with 1 μM; WKYMVm for another 15 min and fixed. After staining the actin and nuclei, the results showed that C18 indeed interfered with morphological changes induced by FPR2 agonists. It could limit cell extrusion induced by WKYMVm (**Figure 5**), but had no effect on unstimulated RBL-FPR2 cells. Therefore, C18 exerts an effect through FPR2. This may also explain why C18 could inhibit cell chemotaxis towards FPR2 agonist, but the detailed mechanism still need to be further investigated.

### Comparison of FPR2 Conformational Changes Induced by C18 and WKYMVm

**Figure 6:**
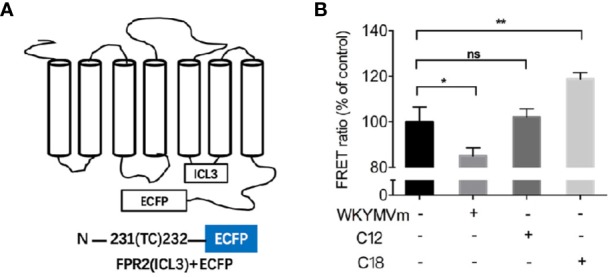
FRET-based measurement of conformational changes of FPR2. Schematic drawing of plasmid coding for FPR2-ICL3-ECFP **(A)**. FPR2 conformational changes induced by WKYMVm (1 μM) or C18 or C12 (10 μM each) and shown as changes in FRET ratio **(B)**. FPR2 construct with a ECFP at the C-terminal and a FLAsH-binding sequence in the third intracellular loop was transiently transfected into HEK-293 cells. Twenty-four hours after transfection, the cell were washed with HBSS buffer and incubated with FlAsH-EDT2 labeling reagent for 1 h. After removing the excess and nonspecifically bound FLAsH, cells were then treated with or without the indicated ligands for 10 min. The coverslips with the cells were mounted on glass slides and FRET signals were analyzed immediately using a Leica TCS SP8 confocal microscope. The emission paths are set at 535 ± 15 nm (FLAsH) and 480 ± 20 nm (ECFP), respectively. FRET signals were calculated as ratio of FLAsH intensities to ECFP intensities based on the corresponding two images. Samples (n = 5 with 5 × 5 pixels each) were collected for fluorescent intensity analysis. Data are shown as Mean ± SD of 5 samples taken from three independent experiments, each with similar results. ^*^P< 0.05; ^**^P< 0.01 versus vehicle-treated cells (control group). FRET, FLAsH-based Fluorescence Resonance Energy Transfer; ECFP, enhanced cyan fluorescent protein; HBSS, Hank's balanced salt solution; NS, No significance.

Considering that C18 could compete for the active binding site of FPR2, FRET detection assay was conducted to investigate molecular dynamics of ligand-induced receptor conformation. FPR2 fluorescent biosensors were generated by placing into one of its intracellular loops a FlAsH binding motif and an enhanced cyan fluorescent protein (ECFP) in its C-terminus (**Figure 6A**). Using this FPR2(ICL3) + ECFP construct, WKYMVm induced a decrease in FRET signal, suggesting that the C-terminal ECFP moved away from the ICL3-inserted FlAsH (**Figure 6B**). In comparison, C18 but not C12 induced an opposite conformational change, suggesting that the C-terminal ECFP moved closer to the ICL3 upon C18 stimulation. These results confirmed that C18 could act directly on FPR2 and cause conformation changes opposite to those induced by WKYMVm, thereby reducing the stimulation effects. The fact that C12 could not induced conformational changes of FPR2 indicates selectivity of C18 at the receptor level.

### Analysis of the Interaction Between C18 and FPR2 With Molecular Docking

**Figure 7:**
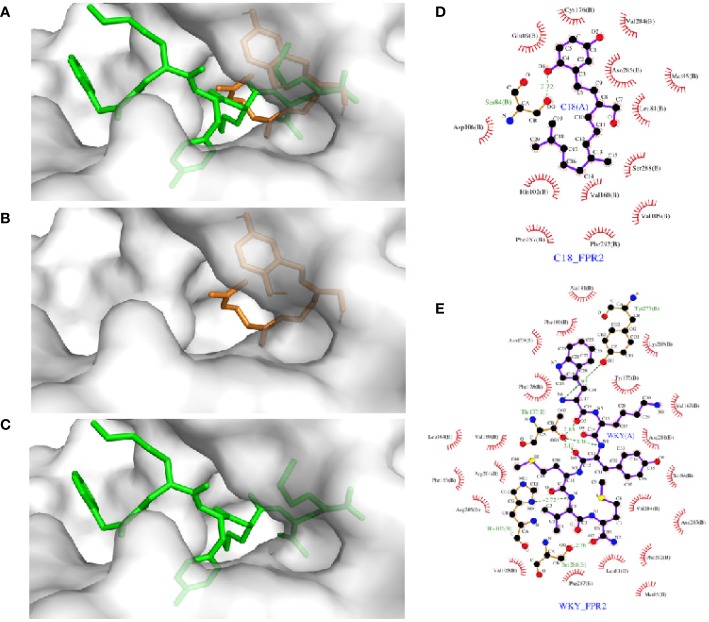
Molecular docking analysis of the interaction between FPR2 and its ligands, C18, and WKYMVm. Docking models of FPR2 was obtained from Swiss-model server which was based on C5a receptor (PDB code: 5o9hA) because of its higher sequence similarity (34.5%) and resolution (2.7 Å). The initial conformations of the ligands were generated by ChemBio 3D. Hydrogens were added by Autodock Tools and molecular docking was performed by Autodock Vina. The search box was set as 46 Å × 34 Å × 60 Å for FPR2. The best conformation was refined with energy minimization and analyzed with PyMOL molecular graphics system. The docking results show that FPR2 has a relatively large binding pocket **(A)**, and C18 could occupy a part of the FPR2 binding pocket **(B)** compared with WKYMVm **(C)** (C18 is colored in orange, WKYMVm in green). There was a partial overlap between the C18 binding site and the WKYMVm binding site in FPR2. C18 is predicted to form hydrogen bond with Ser^84^ and several hydrophobic interactions with FPR2, such as Leu^81^, Met^85^, Ser^288^, Val^160^, His^102^, Phe^257^, and probably more **(D)**. WKYMVm is predicted to form six hydrogen bonds with His^102^, Thr^177^, Tyr^277^, and Ser^288^ and several hydrophobic interactions **(E)**. FPR2, Formyl peptide receptors.

Molecular docking analysis was performed to investigate the detailed interaction between C18 and FPR2. As shown in **Figure 7**, FPR2 has a relatively large binding pocket for its agonists. The pan-agonist WKYMVm has a high calculated affinity for FPR2 and fits well with this C5a-based model by occupying the binding pocket completely. Compared with WKYMVm, C18 could only occupy a small area of the binding pocket with a lower calculated affinity, and the affinity was also confirmed by previous competitive binding assay (**Figure 4C**). It was predicted that C18 forms one key hydrogen bond with Ser^84^ and several hydrophobic interactions with FPR2. The molecular binding of C18 and FPR2, based on the docking analysis, blocks some of the binding sites of WKYMVm, such as Ser^84^, His^102^,Val^105^, Phe^257^, Asn^285^, Phe^292^, that are vital to ligand binding and hydrogen bond formation for FPR2 (Fujita et al., 2011; Schepetkin et al., 2011; Stepniewski and Filipek, 2015). The docking data provide a structural basis for C18 interaction with FPR2.

## Discussion

As FPRs are important regulators in various disease, low-molecular-weight compounds that could target FPRs and FPR-related signaling pathways may have great potential in the discovery of drugs for treating inflammatory diseases (Dahlgren et al., 2016). Several FPR-mediated functional assays were adopted for screening of *Ganoderma*-derived compounds and one compound, C18, was found to clearly limit the FPR2 agonist-induced cellular responses. Further studies investigated the molecular mechanism under these immune-modulating actions, and the results showed that C18 could inhibit FPR-mediated cell extrusion as well as superoxide generation. Meanwhile C18 caused FPR2 conformational changes that are different from the agonist induced conformational changes. Since C18 could compete partially with WKYMVm at higher concentration, it perhaps binds to an allosteric site on FPR2 and then partially limits FPR-mediated cellular responses. The assumption was further confirmed by molecular docking analysis. C18 could form one hydrogen bond and several hydrophobic interactions with FPR2. Based on these results, it was suggested that the *Ganderma*-derived C18 compound could exert its inhibitory effects through binding to an allosteric site on FPR2. The binding causes a conformational change that limit the activation of the receptor by its agonist, and further inhibit its downstream signaling pathway, resulting in reduced production of superoxide anions and compromised cell chemotaxis, thus relieving the symptoms of inflammation. Based on these results, we believed that *Ganoderma*-derived C18 may be a potential candidate for anti-inflammatory compound that exerts inhibitory effects through FPR2.

It is well documented that herbal ingredients, including many that exhibit anti-inflammatory activities, can act on multiple targets. To identify the potential target (s) of actions of C18, we conducted a number of assays including competitive binding and differential activation of the NADPH oxidase through FPR-dependent (fMLF and WKYMVm) and FPR-independent (PMA) pathways. In addition, we have included a different chemoattractant receptor, the C5a receptor, that uses similar or even identical signaling pathways for the activation of cellular functions in neutrophils (Rabiet et al., 2007). Our results clearly demonstrate that C18 inhibited the FPR-mediated superoxide generation while having no effects on C5a receptor-mediated and PKC-dependent (PMA-induced) superoxide activation. C18 was not as effective on FPR1-mediated superoxide generation, prompting us to select FPR2 for further investigation on conformational changes induced by WKYMVm, C12 and C18. Using a FLAsH-based single-molecule FRET detection assay, we found that WKYMVm and C18 altered the FRET intensity in opposite directions, suggesting that C18 interacts directly with FPR2 and induces different conformational changes in FPR2. In comparison, C12, a structural analogue of C18, failed to induce FPR2 conformational changes suggesting that C18 is highly selective for FPR2. Combined with the results from the competitive binding assay that showed lower binding affinity, we postulate that C18 serves as a negative modulator of FPR2 in a manner that differs from a typical neutral antagonist.

Due to the absence of crystal structures for FPRs, the detailed recognition between FPRs and ligands remains unclear. To explain ligand-FPR interactions, homology models were adopted and molecular docking analysis based on the previously site-directed mutagenesis studies were used as an alternative method. In this study, the recently acquired crystal structure of C5a receptor was used as a template model due to its higher sequence similarity (34.5%) and resolution (2.7 Å) than the previously used CXCR4 template (He and Ye, 2017). The C5a receptor-based model revealed a relatively larger binding pocket of FPR2, and the natural agonist WKYMVm fits well with this model. More recently, Stepniewski et al. used a dual template approach with CXCR4 and μOR, and not only confirmed the previously identified residues such as His^102^, Phe^257^, and Arg^201^ for ligand binding, but also found two novel residues, Ser^84^ and Asn^285^, presumably important for the formation of hydrogen bonds (Stepniewski and Filipek, 2015). In the present study, we found that C18 could form hydrogen bond with Ser^84^, while other structural homologs such as C12 could not form hydrogen bond with Ser^84^ (data not shown). This amino acid will be of interest in future studies using site-directed mutagenesis. C18 contains more hydroxyl groups (-OH), that may be acceptors or donors of H-bonding, than its analogues in this group of *Ganoderma*-derived compounds. This H-bonding donor/acceptor feature indicates that C18 has similar property as those of the reported FPRs antagonists (Schepetkin et al., 2014), that inhibit FPR-mediated pro-inflammatory response.

Our initial experiments were conducted using dHL60 cells that express both FPR1 and FPR2. However, when using stably-transfected FPR1-RBL cells, C18 had no effect on maximal Ca^2+^ mobilization induced by fMLF, not did it compete with FITC-labelled fMLF (data not shown). These and other results shown in this study support the notion that C18 acts at FPR2. Since FPR1 and FPR2 can form homodimer or heterodimer as reported by Cooray and coworkers (Cooray et al., 2013), each dimer of FPR may yield specific signaling pathways to resolve inflammation (Filep, 2013; Krishnamoorthy et al., 2018). Thus, C18 should be further tested in cells for the possibility of using FPR heterodimer (FPR1-FPR2) or homodimer (FPR2-FPR2) for potential anti-inflammatory activity.

As a new compound of *Ganoderma* meroterpenoids (GMs), C18 was firstly isolated in 2016, and the molecular formula was determined as C_21_H_30_O_3_ by HRESIMS and ^13^C-DEPT NMR (Peng et al., 2016). Although the chemical properties have been determined, its biological activities remain unclear. GMs include two parts, a 1,2,4-trisubstituted phenyl and a polyunsaturated terpenoid. Compared the structure of these GMs in **Figure 1**, it is evident that the hydroxyl group on polyunsaturated terpenoid plays a vital role in bioactivities. These diverse structural skeletons and related bioactivities of GMs, as well as the development of chemical synthesis methods (Peng and Qiu, 2018), have attracted more attention in recently years. Considering that the IC_50_ of C18 is still poor, there is a need for further modification of the compound for better activity and reduced cytotoxicity, that will be used for investigation of the anti-inflammatory effects with *in vivo* studies.

## Data Availability Statement

The raw data supporting the conclusions of this article will be made available by the authors, without undue reservation, to any qualified researcher.

## Author Contributions

HW and XP contributed equally to this work. HW performed experiments, collected and analyzed data, and prepared figures. XP purified compounds and performed initial characterization. YG and SZ prepared fluorescent biosensors and assisted in functional assays. YF performed supplementary chemotaxis assay. ZW and WH performed molecular docking and analysis. MQ and RY designed the study. RY and HW wrote the manuscript. All authors have given approval to the final version of the manuscript.

## Funding

This work was supported by funds from the University of Macau (MYRG2016-00152-ICMS-QRCM and MYRG2016-00246-ICMS-QRCM) and from Southern University of Science and Technology (Guangdong-Hong Kong joint innovation Research Scheme 2016A050503010 and Shenzhen Peacock Plan Project KQTD2016053117035204).

## Conflict of Interest

The authors declare that the research was conducted in the absence of any commercial or financial relationships that could be construed as a potential conflict of interest.

## Abbreviations

FPRs, Formyl peptide receptors; GPCRs, G protein-coupled receptors; fMLF, N-Formyl-Met-Leu-Phe; PMA, phorbol-12-myristate-13-acetate; C5a, complement component 5a; MFI, mean fluorescent intensity; FRET, FLAsH-based Fluorescence resonance energy transfer; dHL60, differential HL-60; PKC, protein kinase C; DAG, diacylglycerol; BSA, Bovine Serum Albumin; ECFP, Enhanced Cyan Fluorescent Protein; GMs, *Ganoderma* meroterpenoids.

## References

[B1] Baracco E. E. Pietrocola F. Buque A. Bloy N. Senovilla L. Zitvogel L. (2016). Inhibition of formyl peptide receptor 1 reduces the efficacy of anticancer chemotherapy against carcinogen-induced breast cancer. Oncoimmunology 5 (6), e1139275. 10.1080/2162402X.2016.1139275 27471610 PMC4938360

[B2] Braun M. C. Wang J. M. Lahey E. Rabin R. L. Kelsall B. L. (2001). Activation of the formyl peptide receptor by the HIV-derived peptide T-20 suppresses interleukin-12 p70 production by human monocytes. Blood 97 (11), 3531–3536. 10.1182/blood.v97.11.3531 11369647

[B3] Cheng T. Y. Wu M. S. Lin J. T. Lin M. T. Shun C. T. Hua K. T. (2014). Formyl Peptide receptor 1 expression is associated with tumor progression and survival in gastric cancer. Anticancer Res. 34 (5), 2223–2229. 24778024

[B4] Claria J. Dalli J. Yacoubian S. Gao F. Serhan C. N. (2012). Resolvin D1 and resolvin D2 govern local inflammatory tone in obese fat. J. Immunol. 189 (5), 2597–2605. 10.4049/jimmunol.1201272 22844113 PMC3424332

[B5] Cooray S. N. Gobbetti T. Montero-Melendez T. McArthur S. Thompson D. Clark A. J. L. (2013). Ligand-specific conformational change of the G-protein-coupled receptor ALX/FPR2 determines proresolving functional responses. Proc. Natl. Acad. Sci. U. S. A. 110 (45), 18232–18237. 10.1073/pnas.1308253110 24108355 PMC3831442

[B6] Dahlgren C. Karlsson A. (1999). Respiratory burst in human neutrophils. J. Immunol. Methods 232 (1), 3–14. 10.1016/S0022-1759(99)00146-5 10618505

[B7] Dahlgren C. Gabl M. Holdfeldt A. Winther M. Forsman H. (2016). Basic characteristics of the neutrophil receptors that recognize formylated peptides, a danger-associated molecular pattern generated by bacteria and mitochondria. Biochem. Pharmacol. 114, 22–39. 10.1016/j.bcp.2016.04.014 27131862

[B8] De Paulis A. Prevete N Fiorentino I. Walls A. F. Curto M. Petraroli A. (2004). Basophils infiltrate human gastric mucosa at sites of Helicobacter pylori infection, and exhibit chemotaxis in response to H. pylori-derived peptide Hp (2–20). J. Immunol. 172 (12), 7734–7743. 10.4049/jimmunol.172.12.7734 15187157

[B9] Dorward D. A. Lucas C. D. Chapman G. B. Haslett C. Rossi A. G. (2015). The role of formylated peptides and formyl peptide receptor 1 in governing neutrophil function during acute inflammation. Am. J. Pathol. 185 (5), 1172–1184. 10.1016/j.ajpath.2015.01.020 25791526 PMC4419282

[B10] Filep J. G. (2013). Biasing the lipoxin A(4)/formyl peptide receptor 2 pushes inflammatory resolution. Proc. Natl. Acad. Sci. U. S. A. 110 (45), 18033–18034. 10.1073/pnas.1317798110 24154723 PMC3831470

[B11] Fujita H. Kato T. Watanabe N. Takahashi T. Kitagawa S. (2011). Stimulation of human formyl peptide receptors by calpain inhibitors: homology modeling of receptors and ligand docking simulation. Arch. Biochem. Biophys. 516 (2), 121–127. 10.1016/j.abb.2011.09.017 22005393

[B12] Gaietta G. Deerinck T. J. Adams S. R. Bouwer J. Tour O. Laird D. W. (2002). Multicolor and electron microscopic imaging of connexin trafficking. Science 296 (5567), 503–507. 10.1126/science.1068793 11964472

[B13] Gao Y. Zhou S. Huang M. Xu A. (2003). Antibacterial and antiviral value of the genus Ganoderma P. Karst. species (Aphyllophoromycetideae): a review. Int. J. Med. Mushrooms 5 (3), 235–246. 10.1615/InterJMedicMush.v5.i3.20

[B14] Hauert A. B. Martinelli S. Marone C. Niggli V. (2002). Differentiated HL-60 cells are a valid model system for the analysis of human neutrophil migration and chemotaxis. Int. J. Biochem. Cell Biol. 34 (7), 838–854. 10.1016/S1357-2725(02)00010-9 11950599

[B15] He H. Q. Ye R. D. (2017). The formyl peptide receptors: diversity of ligands and mechanism for recognition. Molecules 22 (3), pii: E455. 10.3390/molecules22030455 28335409 PMC6155412

[B16] He R. Tan L. Browning D. D. Wang J. M. Ye R. D. (2000). The synthetic peptide Trp-Lys-Tyr-Met-Val-D-Met is a potent chemotactic agonist for mouse formyl peptide receptor. J. Immunol. 165 (8), 4598–4605. 10.4049/jimmunol.165.8.4598 11035102

[B17] He H. Q. Liao D. Wang Z. G. Wang Z. L. Zhou H. C. Wang M. W. (2013). Functional characterization of three mouse formyl peptide receptors. Mol. Pharmacol. 83 (2), 389–398. 10.1124/mol.112.081315 23160941 PMC4170117

[B18] Hoffmann C. Gaietta G. Bunemann M. Adams S. R. Oberdorff-Maass S. Behr B. (2005). A FlAsH-based FRET approach to determine G protein-coupled receptor activation in living cells. Nat. Methods 2 (3), 171–176. 10.1038/nmeth742 15782185

[B19] Joo S. S. Ryu I. W. Park J. K. Yoo Y. M. Lee D. H. Hwang K. W. (2008). Molecular cloning and expression of a laccase from Ganoderma lucidum, and its antioxidative properties. Mol. Cells 25 (1), 112–118. 18319622

[B20] Kao W. Gu R. Jia Y. Wei X. Fan H. Harris J. (2014). A formyl peptide receptor agonist suppresses inflammation and bone damage in arthritis. Br. J. Pharmacol. 171 (17), 4087–4096. 10.1111/bph.12768 24824742 PMC4243981

[B21] Krishnamoorthy N. Abdulnour R. E. E. Walker K. H. Engstrom B. D. Levy B. D. (2018). Specialized proresolving mediators in innate and adaptive immune responses in airway diseases. Physiol. Rev. 98 (3), 1335–1370. 10.1152/physrev.00026.2017 29717929 PMC6168922

[B22] Li B. Q. Wetzel M. A. Mikovits J. A. Henderson E. E. Rogers T. J. Gong W. (2001). The synthetic peptide WKYMVm attenuates the function of the chemokine receptors CCR5 and CXCR4 through activation of formyl peptide receptor-like 1. Blood 97 (10), 2941–2947. 10.1182/blood.V97.10.2941 11342415

[B23] Li S. Q. Su N. Gong P. Zhang H. B. Liu J. Wang D. (2017). The expression of formyl peptide receptor 1 is correlated with tumor invasion of human colorectal cancer. Sci. Rep. 7 (1), 5918. 10.1038/s41598-017-06368-9 28724995 PMC5517416

[B24] Luo Q. Wang X. L. Di L. Yan Y. M. Lu Q. Yang X. H. (2015). Isolation and identification of renoprotective substances from the mushroom Ganoderma lucidum. Tetrahedron 71 (5), 840–845. 10.1016/j.tet.2014.12.052

[B25] Ma J. Liu C. Chen Y. Jiang J. Qin Z. (2011). Cellular and molecular mechanisms of the Ganoderma applanatum extracts induces apoptosis on SGC-7901 gastric cancer cells. Cell Biochem. Funct. 29 (3), 175–182. 10.1002/cbf.1735 21465494

[B26] Mellor H. Parker P. J. (1998). The extended protein kinase C superfamily. Biochem. J. 332 ( Pt 2), 281–292. 10.1042/bj3320281 9601053 PMC1219479

[B27] Migeotte I. Communi D. Parmentier M. (2006). Formyl peptide receptors: a promiscuous subfamily of G protein-coupled receptors controlling immune responses. Cytokine Growth Factor Rev. 17 (6), 501–519. 10.1016/j.cytogfr.2006.09.009 17084101

[B28] Murphy P. M. Ozcelik T. Kenney R. T. Tiffany H. L. McDermott D. Francke U. (1992). A structural homologue of the N-formyl peptide receptor. Characterization and chromosome mapping of a peptide chemoattractant receptor family. J. Biol. Chem. 267 (11), 7637–7643. 1373134

[B29] Nanamori M. Cheng X. Mei J. Sang H. Xuan Y. Zhou C. (2004). A novel nonpeptide ligand for formyl peptide receptor-like 1. Mol. Pharmacol. 66 (5), 1213–1222. 10.1124/mol.104.004309 15308762

[B30] Nie B. Cheng N. Dinauer M. C. Ye R. D. (2010). Characterization of P-Rex1 for its role in fMet-Leu-Phe-induced superoxide production in reconstituted COS(phox) cells. Cell Signal 22 (5), 770–782. 10.1016/j.cellsig.2010.01.001 20074642 PMC3282168

[B31] Odobasic D. Jia Y. Kao W. Fan H. Wei X. Gu R. (2018). Formyl peptide receptor activation inhibits the expansion of effector T cells and synovial fibroblasts and attenuates joint injury in models of rheumatoid arthritis. Int. Immunopharmacol. 61, 140–149. 10.1016/j.intimp.2018.05.028 29879657

[B32] Park Y. J. Park B. Lee M. Jeong Y. S. Lee H. Y. Sohn D. H. (2018). A novel antimicrobial peptide acting via formyl peptide receptor 2 shows therapeutic effects against rheumatoid arthritis. Sci. Rep. 8 (1), 14664. 10.1038/s41598-018-32963-5 30279454 PMC6168567

[B33] Park G. T. Kwon Y. W. Lee T. W. Kwon S. G. Ko H. C. Kim M. B. (2019). Formyl peptide receptor 2 activation ameliorates dermal fibrosis and inflammation in Bleomycin-induced Scleroderma. Front. Immunol. 10, 2095. 10.3389/fimmu.2019.02095 31552041 PMC6733889

[B34] Peng X. Qiu M. (2018). Meroterpenoids from Ganoderma species: a review of last five years. Nat. Prod. Bioprospect 8 (3), 137–149. 10.1007/s13659-018-0164-z 29722004 PMC5971034

[B35] Peng X. Li L. Wang X. Zhu G. Li Z. Qiu M. (2016). Antioxidant farnesylated hydroquinones from Ganoderma capense. Fitoterapia 111, 18–23. 10.1016/j.fitote.2016.04.006 27083379

[B36] Peng X. Wang X. Chen L. Yang H. Li L. Lu S. (2018). Racemic meroterpenoids from Ganoderma cochlear. Fitoterapia 127, 286–292. 10.1016/j.fitote.2018.03.005 29540310

[B37] Prevete N. Liotti F. Visciano C. Marone G. Melillo R. M. de Paulis A. (2015). The formyl peptide receptor 1 exerts a tumor suppressor function in human gastric cancer by inhibiting angiogenesis. Oncogene 34 (29), 3826–3838. 10.1038/onc.2014.309 25263443

[B38] Prevete N. Liotti F. Illiano A. Amoresano A. Pucci P. de Paulis A. (2017). Formyl peptide receptor 1 suppresses gastric cancer angiogenesis and growth by exploiting inflammation resolution pathways. Oncoimmunology 6 (4), e1293213. 10.1080/2162402X.2017.1293213 28507800 PMC5414878

[B39] Price M. O. McPhail L. C. Lambeth J. D. Han C. H. Knaus U. G. Dinauer M. C. (2002). Creation of a genetic system for analysis of the phagocyte respiratory burst: high-level reconstitution of the NADPH oxidase in a nonhematopoietic system. Blood 99 (8), 2653–2661. 10.1182/blood.V99.8.2653 11929750

[B40] Rabiet M. J. Huet E. Boulay F. (2007). The N-formyl peptide receptors and the anaphylatoxin C5a receptors: an overview. Biochimie 89 (9), 1089–1106. 10.1016/j.biochi.2007.02.015 17428601 PMC7115771

[B41] Robertson N. Rappas M. Dore A. S. Brown J. Bottegoni G. Koglin M. (2018). Structure of the complement C5a receptor bound to the extra-helical antagonist NDT9513727. Nature 553 (7686), 111–114. 10.1038/nature25025 29300009

[B42] Sanodiya B. S. Thakur G. S. Baghel R. K. Prasad G. B. Bisen P. S. (2009). Ganoderma lucidum: a potent pharmacological macrofungus. Curr. Pharm. Biotechnol. 10 (8), 717–742. 10.2174/138920109789978757 19939212

[B43] Schepetkin I. A. Kirpotina L. N. Khlebnikov A. I. Jutila M. A. Quinn M. T. (2011). Gastrin-releasing peptide/neuromedin B receptor antagonists PD176252, PD168368, and related analogs are potent agonists of human formyl-peptide receptors. Mol. Pharmacol. 79 (1), 77–90. 10.1124/mol.110.068288 20943772 PMC3014281

[B44] Schepetkin I. A. Khlebnikov A. I. Giovannoni M. P. Kirpotina L. N. Cilibrizzi A. Quinn M. T. (2014). Development of small molecule non-peptide formyl peptide receptor (FPR) ligands and molecular modeling of their recognition. Curr. Med. Chem. 21 (13), 1478–1504. 10.2174/0929867321666131218095521 24350845 PMC6791360

[B45] Sliva D. (2004). Cellular and physiological effects of Ganoderma lucidum (Reishi). Mini Rev. Med. Chem. 4 (8), 873–879. 10.2174/1389557043403323 15544548

[B46] Stepniewski T. M. Filipek S. (2015). Non-peptide ligand binding to the formyl peptide receptor FPR2—A comparison to peptide ligand binding modes. Bioorganic Med. Chem. 23 (14), 4072–4081. 10.1016/j.bmc.2015.03.062 25882522

[B47] Su L. D. Peng J. M. Ge Y. B. (2018). Formyl peptide receptor 2 mediated chemotherapeutics drug resistance in colon cancer cells. Eur. Rev. Med. Pharmacol. Sci. 22 (1), 95–100. 10.26355/eurrev_201801_14105 29364475

[B48] Svensson L. Redvall E. Björn C. Karlsson J. Bergin A. M. Rabiet M. J. (2007). House dust mite allergen activates human eosinophils via formyl peptide receptor and formyl peptide receptor-like 1. Eur. J. Immunol. 37 (7), 1966–1977. 10.1002/eji.200636936 17559171

[B49] Trott O. Olson A. J. (2010). AutoDock Vina: improving the speed and accuracy of docking with a new scoring function, efficient optimization, and multithreading. J. Comput. Chem. 31 (2), 455–461. 10.1002/jcc.21334 19499576 PMC3041641

[B50] Waterhouse A. Bertoni M. Bienert S. Studer G. Tauriello G. Gumienny R. (2018). SWISS-MODEL: homology modelling of protein structures and complexes. Nucleic Acids Res. 46 (W1), W296–W303. 10.1093/nar/gky427 29788355 PMC6030848

[B51] Weiss E. Kretschmer D. (2018). Formyl-peptide receptors in infection, inflammation, and cancer. Trends Immunol. 39 (10), 815–829. 10.1016/j.it.2018.08.005 30195466

[B52] Wollam J. Riopel M. Xu Y. J. Johnson A. M. F. Ofrecio J. M. Ying W. (2019). Microbiota-produced N-Formyl Peptide fMLF promotes obesity-induced glucose intolerance. Diabetes 68 (7), 1415–1426. 10.2337/db18-1307 31010956 PMC6609982

[B53] Xiang Y. Yao X. Chen K. Wang X. Zhou J. Gong W. (2016). The G-protein coupled chemoattractant receptor FPR2 promotes malignant phenotype of human colon cancer cells. Am. J. Cancer Res. 6 (11), 2599–2610. 27904774 PMC5126276

[B54] Xie X. Yang M. Ding Y. Yu L. Chen J. (2017). Formyl peptide receptor 2 expression predicts poor prognosis and promotes invasion and metastasis in epithelial ovarian cancer. Oncol. Rep. 38 (6), 3297–3308. 10.3892/or.2017.6034 29039544 PMC5783575

[B55] Xu Z. Chen X. Zhong Z. Chen L. Wang Y. (2011). Ganoderma lucidum polysaccharides: immunomodulation and potential anti-tumor activities. Am. J. Chin. Med. 39 (1), 15–27. 10.1142/S0192415X11008610 21213395

[B56] Ye R. D. Boulay F. Wang J. M. Dahlgren C. Gerard C. Murphy P. M. (2009). International Union of Basic and Clinical Pharmacology. LXXIII. Nomenclature for the formyl peptide receptor (FPR) family. Pharmacol. Rev. 61 (2), 119–161. 10.1124/pr.109.001578 19498085 PMC2745437

[B57] Yuan H. Ma Q. Ye L. Piao G. (2016). The traditional medicine and modern medicine from natural products. Molecules 21 (5), pii: E559. 10.3390/molecules21050559 27136524 PMC6273146

[B58] Yuen J. W. Gohel M. D. (2005). Anticancer effects of Ganoderma lucidum: a review of scientific evidence. Nutr. Cancer 53 (1), 11–17. 10.1207/s15327914nc5301_2 16351502

