# Supplementary material for: A Ganoderma-Derived Compound Exerts Inhibitory Effect Through Formyl Peptide Receptor 2
Source: Front Pharmacol. 2020 Mar 24;11:337. doi: 10.3389/fphar.2020.00337 (PMC7105723; doi:10.3389/fphar.2020.00337)
Supplement: Supplementary file 1 [file DataSheet_1.pdf]

Supplementary Materials

Supplementary Figure

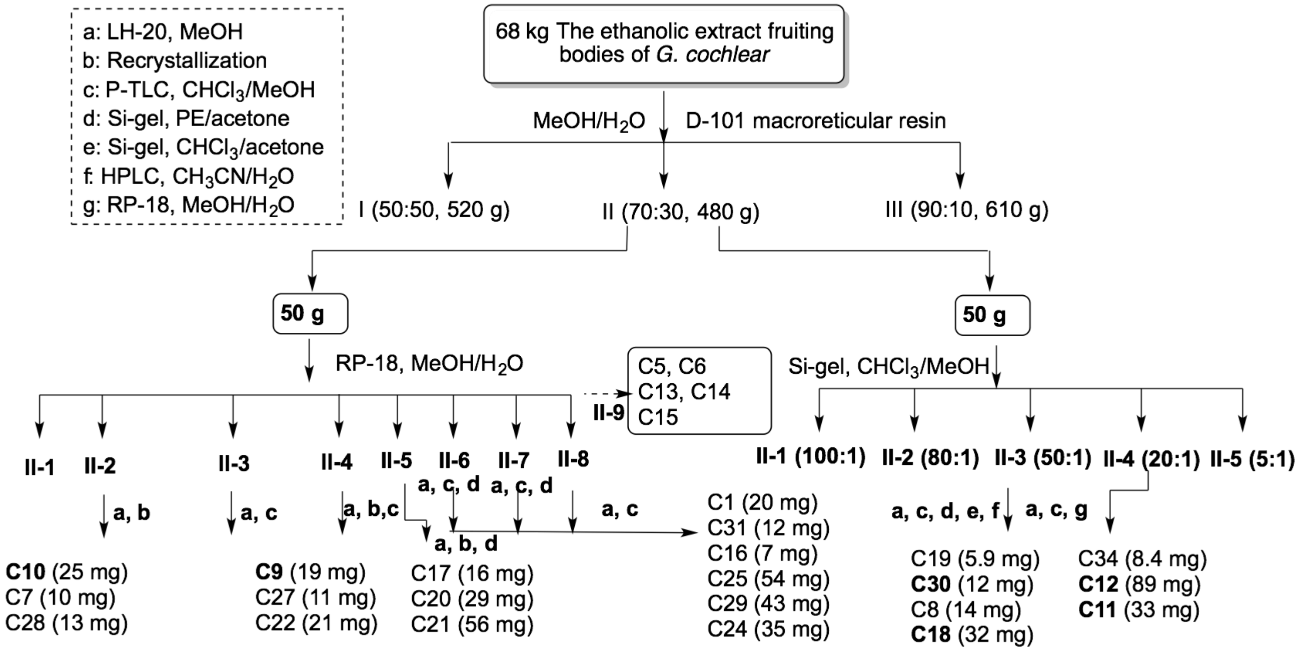

Supplementary Figure 1. *Extraction and isolation of Ganoderma-derived compounds*

A

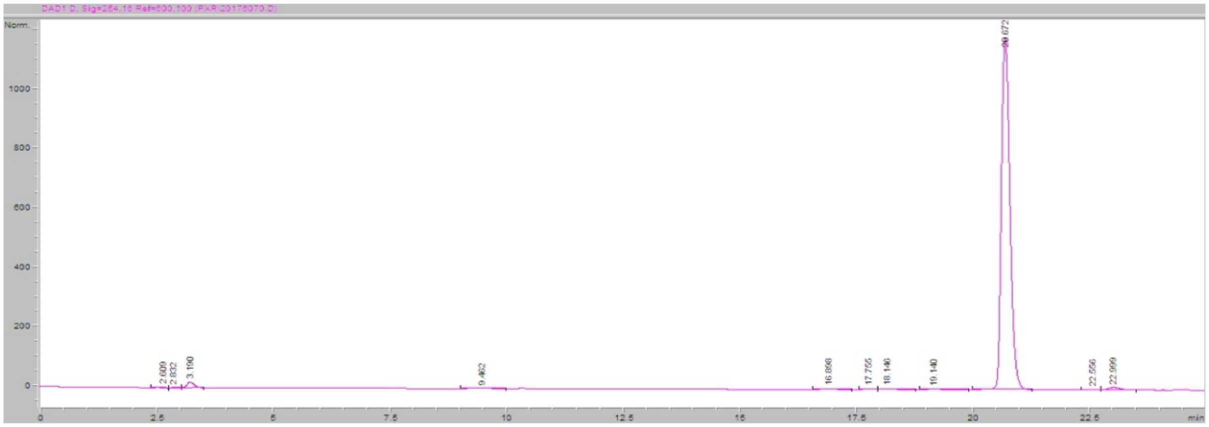

B

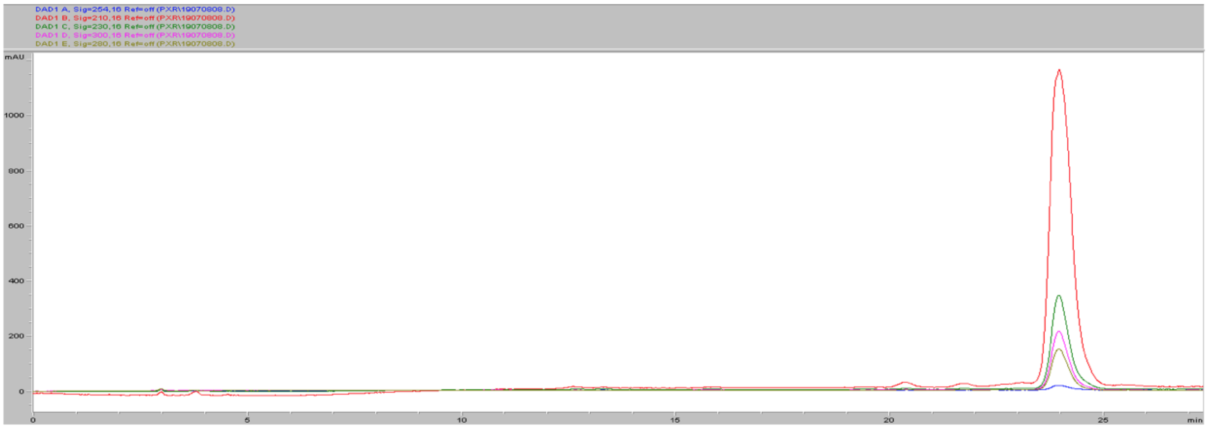

Supplementary Figure 2. *Purity of selected compounds analyzed by HPLC method.*

A. Chromatogram of 254 nm wavelength of C12. C12 was separated by ZORBAX SB-C18 column (acetonitrile-1% acetic acid, 80: 20, t_R = 25 min) and with a purity of 95.4% at 254 nm. **B.** Chromatogram of 210, 230, 254, 280 and 360 nm wavelength of C18. C18 was separated by ZORBAX SB-C18 column (acetonitrile-1% acetic acid, 70: 30, t_R = 27 min) and with a purity of 95% at 210 nm.

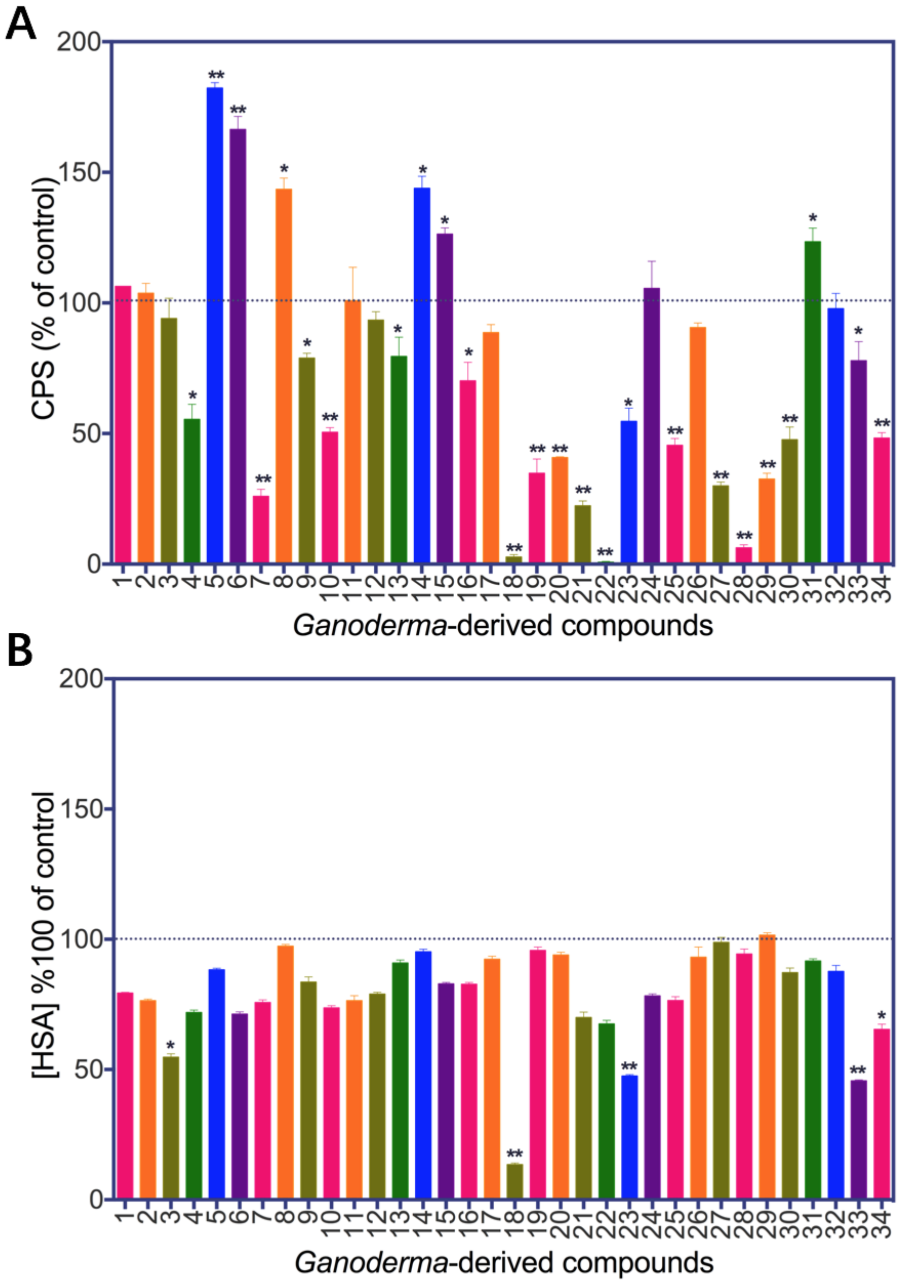

Supplementary Figure 3. *Initial screening results based on differentiated HL-60 cells.*

Differentiated HL60 cells (dHL60-6d, $1×10^5$ cells per well) was pre-incubated with 10 μM *Ganoderma*-derived compounds for 30 min at 37℃, then recorded Chemluminescence per second (CPS) for 25 min after stimulation with 1 μM WKYMVm. Several compounds displayed inhibitory effects based on the superoxide generation assay **(A)**. Pre-incubation dHL60-6d cells ($1×10^5$ cells per well) with 10 μM different *Ganoderma*-derived compounds for 60 min at 37℃, then stimulated with 1 μM WKYMVm to induce β-hexosaminidase (HSA) release **(B)**. Data are shown as Mean ± SD of three independent experiments.

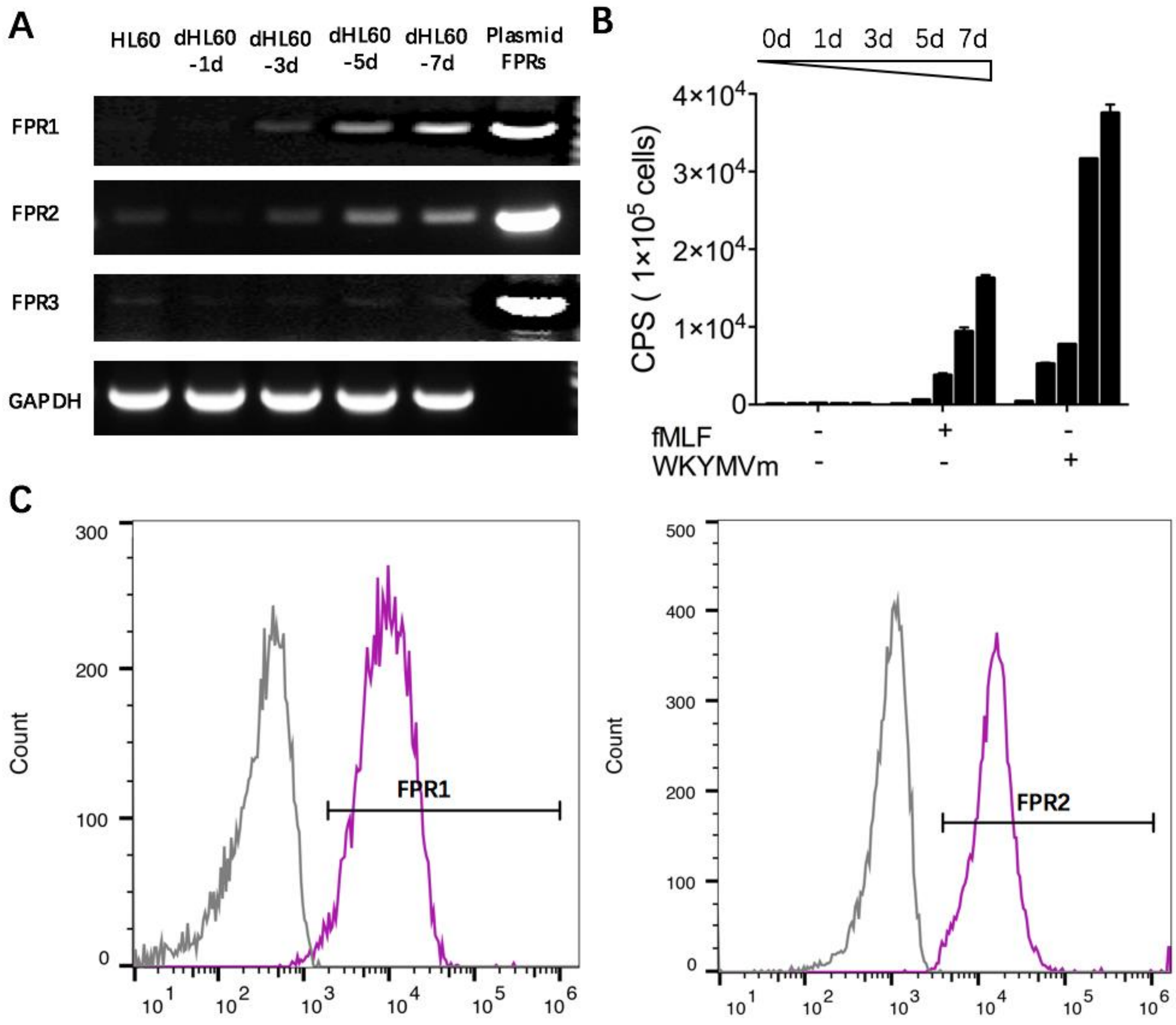

Supplementary Figure 4. *Differentiation of HL-60 cells.*

Human promyelocytic leukemia cell line HL-60 was differentiated with 1.3% DMSO to acquire properties of mature neutrophils. Differentiated HL-60 cells (1, 3, 5, and 7 days) displayed enhanced mRNA expression of FPR1 and FPR2 as determined by RT-PCR **(A)**. The dHL60 cells (differentiated for 1, 3, 5, and 7 days, bars from left to right, 1×10^5 cells per well) showed increased superoxide generation upon stimulation with 1μM fMLF or WKYMVm **(B)**. Protein expression of FPR1 and FPR2 in dHL60-6d cells were examined by flow cytometry with fluorescent labeled anti-FPR1 (left panel) or anti-FPR2 antibody (right panel). HL-60 or dHL60 cells were washed once with PBS (Ca^{2+}, Mg^{2+} free) and blocked 1 hour at room temperature with 5% BSA+PBS (Ca^{2+}, Mg^{2+} free), then stained with FPR1 antibody (Alexa Fluor ®647 mouse anti-human fMLP receptor, Clone 5F1(RUO), BD 565323; 5μL/sample) or FPR2 antibody (NLS 1878, NOVUS; 1:50 for 2×10^5 cells) on ice, 60 min, wash twice with cold PBS. The anti-FPR2 labelled cells were further stained with Molecular probes, Alexa Fluor ®488 goat anti-rabbit IgG (H+L) (A11034, 1:250) on ice, 60 min,

wash twice with cold PBS, then analyzed with cytometry **(C)**. Data shown in (A) and **(C)** are from representative experiments, others are shown as Mean ± SD of three independent experiments.

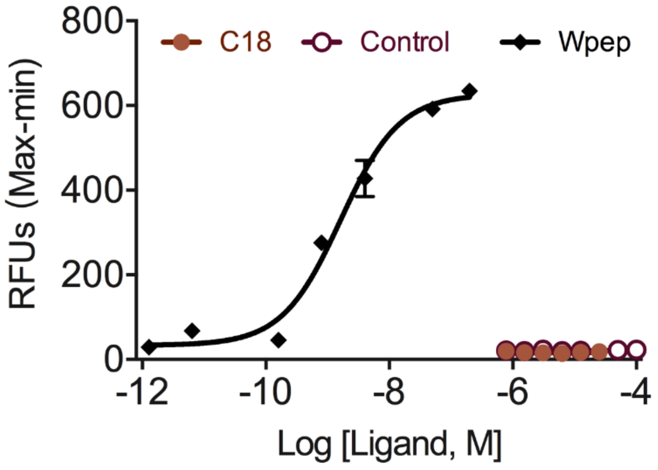

Supplementary Figure 5. *Calcium mobilization induced by C18 and WKYMVm.*

RBL-FPR2 cells were seeded until the confluence reached nearly 90%. After incubation with FLIPR calcium-sensitive dye for 60 min at 37℃, and then stimulated the cells with different concentrations of C18 (1μM - 25 μM) or WKYMVm (10^{-9} - 10^{-4} μM). Relative fluorescence unit (RFU) was recorded. The results show that C18 alone did not induce calcium mobilization compared with WKYMVm. Data are shown as Mean ± SD of three independent experiments.

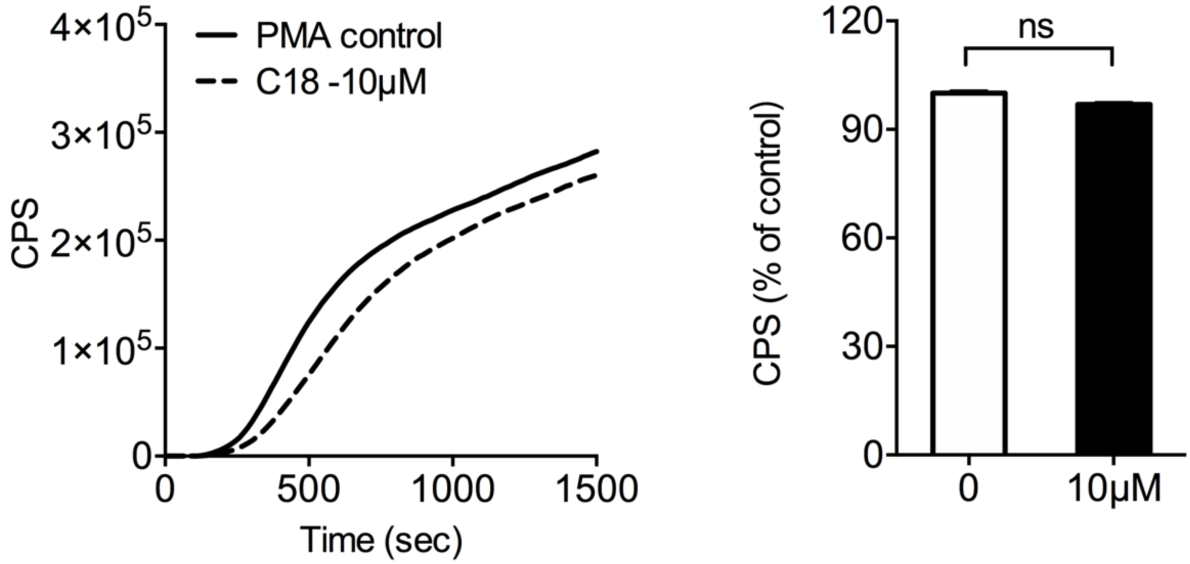

Supplementary Figure 6. *PMA stimulated superoxide generation assay*

Differentiated HL60 cells (dHL60-6d, 1×10^5 cells per well) was pre-incubated with 5 μM compound C18 for 30 min at 37℃, then recorded Chemluminescence per second (CPS) for 25 min after

stimulation with 100 ng/mL PMA. The results show that superoxide generation were not inhibited by compound C18 at 10 μM (C). Data are shown as Mean ± SD of three independent experiments.

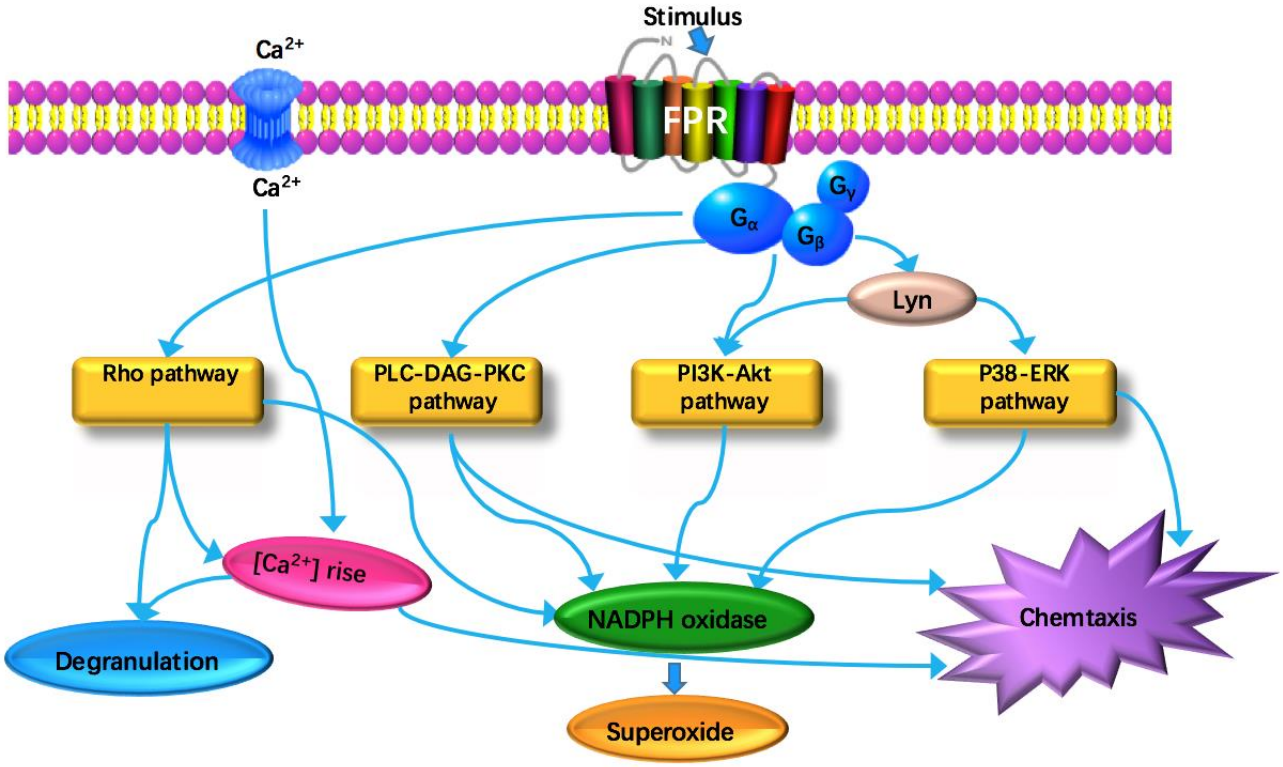

Supplementary Figure 7. *Formyl peptide receptor-mediated signaling pathways.*

Formyl peptide receptors (FPRs) mediate cellular responses including calcium mobilization, superoxide generation, chemotaxis and cell degranulation during phagocyte activation.

Supplementary Table 1. *Checkerboard analysis of C18.*

upper chamber / lower chamber	medium	C18-1μM	C18-10μM	C18-100μM
medium +W pep	51.6±0.9	49.9±2.1	26.2±1.1	0.8±0.1
medium +W pep +C18-1μM	50.1±0.9	47.3±1.0	26.3±0.5	0.5±0.2
medium +W pep +C18-10μM	19.2±1.5	14.±0.6	7.9±0.4	1.3±0.2
medium +W pep +C18-100μM	0.3±0.1	0.4±0.1	0.2±0.0	0.7±0.1

Different concentration of C18 were added to the upper chamber and the lower chamber of transwell plate, and supplemented with 1nM WKYMVm in the lower chamber. Then seeded dHL60 cells (6d, 2×10^5/well) in the upper chamber. After incubation for 2 hours, at 37°. The cells that migrated through the polycarbonate membrane were counted from the lower chamber (Data shown as dHL60 (lower chamber) / total cells $\times100$).
